# Cause of Jaundice

**Published:** 1876-11

**Authors:** 


MANY evidences have been from time to time presented, showing conclusively that jaundice is the result of obstruction to the passage of bile into the bowel. The prevailing opinion is, we believe, that there is an arrest of the biliary secretion, and a consequent retention in the blood of the ingredients from which bile is made. This opinion, however generally entertained, never impressed us as a reasonable theory, for two reasons: 1st, in cases where there is evidently an arrest of secretion, from cirrhosis and other causes, the icterous hue is wanting; 2d, it is acknowledged that bile is a compound made only by the liver, and cannot be deposited in the surface, giving it the yellow tinge, until it has been formed by the gland whose function it is to secrete it. Again, instances of obstruction to the passage of bile into the duodenum, by biliary calculi, have been known, in which yellowness of the skin was a prominent symptom.

Now, in order to sustain a pathological theory of ordinary jaundice, cases of which do not usually admit of post-mortem examinations, practical facts, connected with treatment based on the two theories, are important. Supposing that ordinary icterus, as we find it occurring, sometimes epidemically, is the result of duodinal irritation, congestion and inflammation, the rational plan of treatment would be that of counter-irritation, the soothing influence of opiates and demulcents, and the prevention of increased irritation of the duodenum from acids passing out of the stomach, by mild alkaline preparations. On the other hand, supposing the suppression of bile the cause of jaundice, the rational course is that usually adopted, of exciting the torpid liver with mercurials, etc., which is not successful.

A recent post-mortem proof of the obstruction theory of jaundiced skin will be found in the following :

Jaundice from Atresia of the Bile-duct through Cirrhosis of the Head of the Pancreas.—In the *Annali Universali di Medicinæ Chirurgia* for June, 1876, Dr. Tibaldi describes a case of a married woman, aged thirty-five, who had enjoyed good health up to two months before she was first seen. She had gradually become jaundiced, and had some oppression and pain in the epigastrium and right hypochondrium, with much loss of appetite; she occasionally had slight fever. On examination, Tibaldi found the liver enlarged and resistant; the gall-bladder distended and reaching as far as the umbilicus; the spleen enlarged and resistant, and extending as far as the left iliac region; tympanitic abdomen; regular discharge of colorless fæces; urine scanty, of a yellow-green color; the skin yellow and itching; the impulse and sounds of the heart indistinct; the pulse weak and slow, intermitting at every tenth beat. There was plainly icterus from suppression of the flow of bile. At the necropsy the head of the pancreas was found enlarged to double its normal size, with a mass of puriform matter in its centre, and the bile duct was atrophied for some distance from its point of entrance into the duodenum. Dr. Achilles Visconti found, on histological examination, hyperplasia of the interstitial connective tissue, with pyoid globules and cells of cylindrical epithelium in the pancreatic duct mixed with mucus, without any trace of cancerous formation.—A. Henry, M.D.: *Lond. Med. Rec.*, Sept. 15, 1876.

